# Intraoperative Abdominal Aortic Aneurysm Repair and Its Complications From an Anesthesia Perspective: A Case Report

**DOI:** 10.7759/cureus.37351

**Published:** 2023-04-09

**Authors:** Ahmed K Alanzi, Amir Fouad, Ahmed Mustafa, Hussam Ismail

**Affiliations:** 1 Anesthesia and Critical Care, King Hamad University Hospital, Muharraq, BHR; 2 Radiology, King Hamad University Hospital, Muharraq, BHR

**Keywords:** aaa repair, anesthesia, ultrasound, aortic, abdominal aortic aneurysm

## Abstract

An abdominal aortic aneurysm (AAA) is a disease characterized by an abnormal bulge or swelling in the aorta. It could be serious if left unobserved, and with time, it swells and eventually ruptures, resulting in massive bleeding inside, and, more likely, causes death. This report presents a case study of a 61-year-old male who presented with back pain; no other fatal symptoms such as breathlessness or rapid heart rate were seen. His abdominal ultrasound report showed the presence of a distal aortic dissecting aneurysm, resulting in rapid diagnosis and treatment.

## Introduction

An abdominal aortic aneurysm (AAA) is a segmental, full-thickness dilation of the abdominal aorta that is 50% greater than the normal aortic diameter or abdominal aortic diameter, greater than 3 cm. In general, AAA tends to appear in males more than in females [1]. The diameter varies in the significance of clinical events depending on gender; in males, diameters are mostly predictive of clinical events of the aneurysm, whereas in females, it is more dependent on the aortic size index (ASI), calculated as diameter (cm)/body surface area (m^2^) [2]. AAA is found in 5%-10% of males aged 65-79 [3]. Moreover, the estimated prevalence of AAA in developed countries is 2%-8%, which is higher in males than in females (4%-8% versus 1%-1.3%) [2].

Growth and rupture risk of the aneurysm is associated with female gender, smoking, and chronic lung disease [4]. Elective repair is recommended for aneurysms discovered to be larger than 5.5 cm to prevent rupture [3]. There is interest in population screening to detect, monitor, and repair abdominal aortic aneurysms before rupture [3]. AAA repair can be endovascular or with open surgical repair (OSR) [5], depending on the availability of endovascular repair and surgical team preference.

## Case presentation

A 61-year-old male, with a known case of hypertension, dyslipidemia, and diverticulosis diagnosed more than 20 years ago, presented to the emergency department (ED) with a one-day history of left-sided abdominal pain, which started in the morning. According to the patient, the pain started at 11 am over the left lower back and radiated to the flank and then localized to the left iliac fossa and suprapubic region. His condition was not associated with nausea or vomiting and tolerated oral intake. The patient's bowel was opened that morning at 10:30 am, with no per rectal (PR) bleed, no urinary symptoms, no fever, and no chills or rigors. Severe pain is similar to the previous attacks of his diverticulitis. His past surgical history includes vasectomy and colonoscopy done with polypectomy. He reported smoking a cigar and consuming approximately one bottle of wine five times per week. On arrival to the ED, the patient's temperature was 36.7°C, respiratory rate (RR) was 20 breaths per minute (bpm), random blood glucose was 8 mmol, blood pressure (BP) was 80/50 mmHg, heart rate (HR) was 62 beats per minute (bpm), and oxygen saturation (SpO_2_) was 97%. On examination, his abdomen appeared soft and lax, with tenderness over the left lower abdomen. The requested ECG showed sinus bradycardia with an HR of 48 bpm. Table 1 shows the results of the venous blood gas test.

**Table 1:** Admission laboratory results VBG, venous blood gas; PCO_2_, partial pressure of carbon dioxide; HCO_3_, bicarbonate

Variables	VBG
pH	7.395
PCO_2_	32.1
PO_2_	25.3
HCO_3_	19.2
Hemoglobin	11.74
Sodium (Na)	142.2
Potassium (K)	4.24
Glucose	9.56
Lactate	2.96

At first, he received 1 L of normal saline, and his blood pressure increased to 110/80 mmHg in ED; then, he received paracetamol, diclofenac, fentanyl 50 mcg, and morphine 3 mg. Another 500 ml of normal saline was given. The results from the laboratory initially are showed in Table 2.

**Table 2:** Laboratory findings on admission CRP: C-reactive protein

Variables	Initial results
Creatinine	65.5
Urea	5.1
WBC	15.6
Hemoglobin	12.1
Platelets	225
Sodium	141
Potassium	4.3
CRP	1.1

He was referred to general surgery with the impression of diverticulitis and sepsis.

**Figure 1:**
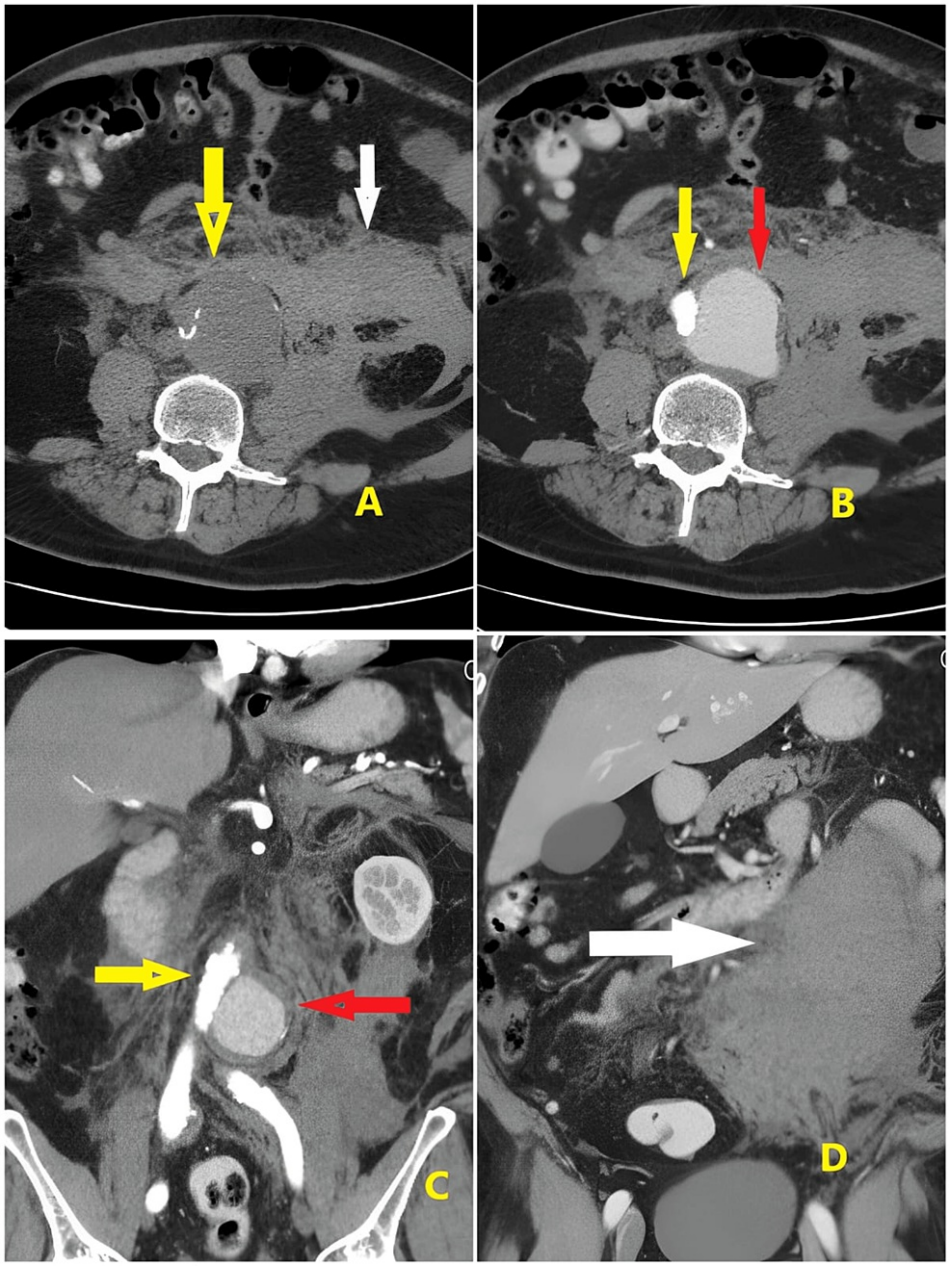
(A) Plain CT of the aorta without contrast (yellow arrow) and surrounding hematoma (white arrow), (B and C) the true lumen (yellow arrow) and the false lumen (red arrow), and (D) a huge retroperitoneal hematoma (white arrow) CT: computed tomography

After the examination, the emergency team planned for computed tomography (CT) of the abdomen, but suddenly, his BP dropped to 60/30 mmHg. He was resuscitated in the ED, and 0.05 mcg/kg norepinephrine was started. Gradually, his BP rose to 100/60 mmHg. His HR was 54 bpm, BP was 102/60 mmHg, RR was 17 bpm, temperature was 36.6°C, and SpO_2_ was 99. The patient's abdomen was re-examined, and tenderness was noted on the left side. Bilateral distal pulses were intact in the lower limbs. The focused assessment with sonography for trauma (FAST) scan showed free fluid in the abdomen. The abdominal X-ray report showed no free air and no air fluid. Phleboliths are noted on the pelvis. An abdominal ultrasound was performed, which revealed an infrarenal ruptured aortic aneurysm with a retroperitoneal bleed. Six units of packed red blood cell (PRBC), six units of platelets, and six units of fresh frozen plasma (FFP) were brought. Abdominal CT report reveals a distal aortic dissecting aneurysm involving the aortic bifurcation and extending to the origin of the left common iliac artery, measuring about 6 cm in maximum length and 6.2 cm in maximum cross-sectional diameter; the true lumen was seen attenuated and compressed to the right side (Figure 1B, yellow arrow), and the false lumen was dilated and faintly filled with contrast at the arterial phase (Figure 1B, red arrow) with more filling at the venous phase measuring about 5.5 × 4.5 cm in maximum axial diameter.

The anterior margin of the psoas muscle is obscured by a blood collection (Figure 1A, 1B, 1D, white arrow). Multiple colonic diverticula were seen more numerous at the sigmoid colon with no acute diverticulitis and patent homogeneously enhanced portal vein and its main divisions. The spleen was average in size, showing normal texture. Both kidneys were of normal size, showing good excretory function; the pancreas was of normal CT appearance and the suprarenal glands and inferior vena cava (IVC). Normal urinary bladder filling showed no stones, masses, or diverticula-enlarged prostate indenting the bladder base. No evidence of enlarged retro-crural, porta-hepatis, or para-aortic lymph nodes was noted.

Lower chest cuts revealed mild bilateral pleural reaction more on the left side. The patient was shifted to the theater at 17:30 for emergency AAA rupture repair, and on arrival to operation theater (OT), the patient had an HR of 115 bpm, BP of 95/50 mmHg, and oxygen saturation of 99%. The patient was intubated with rapid sequence induction and ventilated on pressure-control volume-guaranteed mode. The right internal jugular central line was inserted, 20 G right radial arterial line and 18 G left external jugular line. Right radial arterial line was done after Allen's test, and right jugular central line was done with ultrasound guidance. Etomidate 18 mg and succinylcholine 100 mg were given. After intubation, the patient received atracurium 50 mg. During the procedure, the patient received boluses of epinephrine 100 mcg of a total of 900 mcg, a norepinephrine infusion of 4 mg in 50 ml Ringer's lactate at a rate of 7-24 ml/hour, sodium bicarbonate 100 ml, and unfractionated heparin 5000 IU. Arterial blood gas (ABG) showed a pH of 7.173, partial pressure of carbon dioxide (PCO_2_) of 42, bicarbonate (HCO_3_​)​​​​​​ of 15.6, hemoglobin (Hb) of 6.1, potassium (K) of 4.9, and sodium (Na) of 139. Aortic clamping was done at 20:15; clamping time was approximately two hours. Repeated ABG showed pH of 7.19, Hb of 9.3, PCO_2_ of 38, HCO_3_ of 14.4, K of 4.4, and Na of 137. Total blood loss was 6 L, with total urine output of 500 ml. The input was 8 L of crystalloids, six packed red blood cells (RBCs), and four units of FFP.

The patient was shifted to the intensive care unit (ICU) after repair with an interposition graft. He was first intubated and then sedated on propofol and remifentanil-controlled mandatory ventilation (CMV); his tidal volume (TV) was 450 ml, respiratory rate was 22 bpm, positive end expiratory pressure (PEEP) was 5 cmH_2_O, fraction of inspired oxygen (FiO_2_) was 100%, and BP was 60/40 mmHg on norepinephrine 0.3 mcg/kg/minute. The patient was shifted to OT the next day for revision laparotomy for leaking venous blood and ischemic transverse colon with transverse colectomy and loop colostomy. He was intubated and ventilated, his BP was 56/40 mmHg, he was on noradrenaline infusion of 1 mcg/minute, his SpO_2_ was 88%, and his FiO_2_ was 90%. The patient was sedated with remifentanil 15 mcg/minute and propofol 60 mg/hour, with synchronized intermittent mandatory ventilation (SIMV)-pressure support (PS) mode of ventilation. The patient initially received a noradrenaline infusion of 1 mcg/kg/minute, which was later changed to adrenaline 0.2 mcg/kg/minute as norepinephrine did not respond. Adrenaline 100 mcg bolus was given intermittently in between. The total blood loss was 500 ml, and the patient was anuric. ABG test results are shown in Table 3.

**Table 3:** Arterial blood gas test results ABG, arterial blood gas; PCO_2_, partial pressure of carbon dioxide; HCO_3_, bicarbonate; HCT, hematocrit; SO_2_, sulfur dioxide

Variables	ABG
pH	7.066
PCO_2_	54.5
PO_2_	164.5
HCO_3_	15.3
Hemoglobin	7.40
Sodium	144.7
Potassium	4.29
Barium enema	14.0
HCT	21.4
SO_2_	98.8
Ca_2_	0.938
Glucose	3.81
Lactate	15.00

At the end of the procedure, he was shifted back to the ICU and was intubated and ventilated. His BP was 93/49 mmHg, pulse rate (PR) 90/minute, and SpO_2_ 96% with adrenaline infusion of 0.2 mcg/kg/minute. He developed acute ischemic hepatitis, acute kidney injury, and disseminated intravascular coagulation (DIC). Two days later, his left foot showed bluish discoloration and was cold to touch with absent dorsalis pedis pulsation. And transverse colostomy was malfunctioning. The next day, he was taken to OT for relook laparotomy to explore and revise the rectal stump and left femoral embolectomy by vascular surgery.

He was shifted to OT with unstable hemodynamics. He was given a dopamine supramaximal dose; his SpO_2_ was 85% and FiO_2_ 100% and was put on ventilator. He was started on a noradrenaline infusion of 4 mg in 50 ml at 2-10 ml/hour. The patient was arrested intraoperatively and was resuscitated. Spontaneous circulation returned after three cycles of cardiopulmonary resuscitation (CPR), two doses of adrenaline, and direct cardioversion twice. Ten minutes later, the patient had ventricular tachycardia and received lignocaine 140 mg; synchronized cardioversion was done with 150 J. Sodium bicarbonate 75 ml was given. He received two units of PRBC and was admitted to the ICU. Acute kidney injury on continuous renal replacement therapy (CRRT) disseminated intravascular coagulation. The patient was intubated and sedated for one week with frequent weaning off and reintubation for another week and then extubated. He was shifted to the ward on the June 10, 2020, and was vitally stable on hemodialysis. The patient was discharged after two weeks.

## Discussion

A ruptured abdominal aortic aneurysm request is one of the "hottest" cases on our CT scanner regarding the need for speed. The mortality rate is very high: >90% [6]. Hypertension is considered a potential risk factor that leads toward AAA [7]. Abdominal aortic aneurysm rupture is a surgical emergency. The treatment of an acute rupture should be prompt and can be done with endovascular aneurysm repair or open surgery. It was noted from the retrospective data from the United States that over the past decade, the use of open surgical repair (OSR) has decreased, and a notable increase is observed in the use of endovascular repair for ruptured AAAs [8]. The mortality after rupture is high, 80% for patients reaching the hospital and 50% for those undergoing surgery for emergency repair [2]. In this case, the emergency department team, general and vascular surgeons, anesthetists, and the ICU team resuscitated the patient and provided optimum care. Table 4 shows some similar cases of AAA.

**Table 4:** Demography, clinical characteristics, and laboratory findings of cases of AAA AAA, abdominal aortic aneurysm; BP, blood pressure; CT, computed tomography; RR, respiratory rate; HR, heart rate; bpm, beats per minute; bpm, breaths per minute

Publication year	Age/sex	Presentation	Hemodynamic status	State/size of AAA	Method	Management		Study
2018	68/male	Nausea, hypertension, and increasing lower back pain	BP of 170/115 mmHg	5.8 cm	Abdominal CT scan	Surgical repair of AAA	Petriceks et al. [9]	
2019	79/male	Low back pain radiating to the left side of his chest	BP of 104/66 mmHg, pulse rate of 64/minute, RR of 16/minute, oxygen saturation of 97%, and temperature of 36.5°C	10 cm	Emergent computed tomography angiography (CTA) scan	Open aortic repair (OAR)	Clancy et al. [10]	
2022	58/male	Altered mental state and unilateral extremity weakness	HR of 95 bpm, RR of 17 bpm, BP of 129/76 mmHg, temperature of 96.3°F, and 97% oxygen saturation	Normal in appearance	CTA of the chest, abdomen, and pelvis	Endovascular infrarenal aorta to iliac purification surgery	Garrity et al. [11]	

## Conclusions

Pain is one of the most common symptoms found in patients suffering from AAA, but unfortunately, this is very common, so most people do not seem concerned about it, and before they know about the consequences, it is too late. People should be aware of the importance of screening programs. We suggest that everyone have regular clinical visits and checkups to minimize the chances of such life-threatening events.
